# Clinical features of cluster headache without cranial autonomic symptoms: results from a prospective multicentre study

**DOI:** 10.1038/s41598-021-86408-7

**Published:** 2021-03-25

**Authors:** Min Kyung Chu, Byung-Su Kim, Pil-Wook Chung, Byung-Kun Kim, Mi Ji Lee, Jeong Wook Park, Jin-Young Ahn, Dae Woong Bae, Tae-Jin Song, Jong-Hee Sohn, Kyungmi Oh, Daeyoung Kim, Jae-Moon Kim, Soo-Kyoung Kim, Yun-Ju Choi, Jae Myun Chung, Heui-Soo Moon, Chin-Sang Chung, Kwang-Yeol Park, Soo-Jin Cho

**Affiliations:** 1 Department of Neurology, Severance Hospital, College of Medicine, Yonsei University, Seoul, Korea; 2 Department of Neurology, Bundang Jesaeng General Hospital, Daejin Medical Center, Seongnam, Korea; 3 Department of Neurology, Kangbuk Samsung Hospital, Sungkyunkwan University School of Medicine, Seoul, Korea; 4 Department of Neurology, Eulji Hospital, Eulji University, Seoul, Korea; 5 Department of Neurology, Neuroscience Center, Samsung Medical Center, Sungkyunkwan University School of Medicine, Seoul, Korea; 6 Department of Neurology, Uijeongbu St. Mary’s Hospital, The Catholic University of Korea College of Medicine, Uijeongbu, Korea; 7 Department of Neurology, Seoul, Medical Center, Seoul, Korea; 8 Department of Neurology, St. Vincent’s Hospital, College of Medicine, The Catholic University of Korea, Suwon, Korea; 9 Department of Neurology, College of Medicine, Ewha Womans University Seoul Hospital, Seoul, Korea; 10 Department of Neurology, Chuncheon Sacred Heart Hospital, Hallym University College of Medicine, Chuncheon, Korea; 11 Department of Neurology, Korea University College of Medicine, Seoul, Korea; 12 Department of Neurology, Chungnam National University College of Medicine, Daejeon, Korea; 13 Department of Neurology, Gyeongsang National University College of Medicine, Jinju, Korea; 14 Dr. Choi’s Neurology Clinic, Jeonju, Korea; 15 Department of Neurology, Inje University College of Medicine, Seoul, Korea; 16 Department of Neurology, Chung-Ang University Hospital, Seoul, Korea; 17 Department of Neurology, Dongtan Sacred Heart Hospital, Hallym University College of Medicine, Keun Jae Bong-gil 7, Hwaseong, 18450 Gyeonggi-do Korea

**Keywords:** Medical research, Neurology

## Abstract

Although cranial autonomic symptoms (CAS) are typical in cluster headache (CH), some individuals with CH show no CAS during their headache attacks. Probable cluster headache (PCH) is a subtype of CH that fulfils all but one criterion of CH. This study aimed to investigate the frequency and clinical features of CH and PCH without CAS in comparison to those with CAS. We analysed data from the Korea Cluster Headache Registry, a prospective multicentre registry involving data from 16 hospitals. Of the 216 participants with CH and 26 with PCH, 19 (8.8%) and 7 (26.9%), respectively, did not have CAS. Participants with CH without CAS exhibited less severe anxiety (General Anxiety Disorder-7 score, median [interquartile range], 2.0 [1.0–6.0] vs 8.0 [3.0–12.0], *p* = 0.001) and depression (Patient Health Questionnaire-9 score, 3.0 [1.0–7.0] vs 7.0 [3.0–11.0], *p* = 0.042) than those with CAS. Among participants with PCH, headache intensity was less severe in participants without CAS than in those with CAS (numeric rating scale, 8.0 [7.0–8.0] vs 9.5 [8.0–10.0], *p* = 0.015). In conclusion, a significant proportion of participants with CH and PCH did not have CAS. Some clinical features of CH and PCH differed based on the presence of CAS.

## Introduction

Cluster headache (CH) is characterised by recurrent severe unilateral headache attacks and is accompanied by ipsilateral cranial autonomic symptoms (CAS)^1,2^. The term CH originates from the tendency of headache attacks to cluster during cluster periods that usually last for several weeks to months^3^. The third edition of the International Classification of Headache Disorders (ICHD-3) has divided the CH population into CH (code 3.1) and probable CH (PCH; code 3.5.1)^2^. PCH is a subtype of CH that fulfils all but one of the five criteria for CH and was reported to account for approximately 10–20% of CH cases^2,4,5^. Some clinical features of PCH differ from those of CH. Individuals with PCH have a lower incidence of conjunctival injection and forehead sweating than those with CH, but these groups have showed comparable disability^5,6^.

Although CAS have been recognised as typical symptoms of CH, 3–7% of individuals with CH were noted to never experience CAS during their CH attacks^7–9^. The frequency and clinical features of CH without CAS have been reported in only one instance. A Portuguese study from a single university hospital reported in 2005 that headache intensity was less severe in individuals with CH without CAS than in those with CAS^7^. However, since this study did not distinguish between CH and PCH in its analysis, the frequency and clinical features of CH and PCH without CAS compared to those with CAS remain unclear. Furthermore, this study used data from a single hospital; thus, an additional analysis using data from various settings is needed to validate these findings.

This study aimed to assess (1) the frequencies of CH and PCH without CAS among participants with CH and PCH and (2) the differences in the clinical features of participants with CH and PCH with and without CAS. For this purpose, we used data from the Korean Cluster Headache Registry (KCHR), a prospective, multicentre registry of CH.

## Methods

### Study design and participants

This descriptive, cross-sectional study aimed to investigate the frequency and clinical features of CH and PCH without CAS using data from the KCHR. The KCHR enrolled consecutive participants with CH aged ≥ 19 years from 16 hospitals (14 university hospitals and two secondary referral general hospitals) in Korea. Participants were enrolled between September 2016 and December 2018.

**Figure 1:**
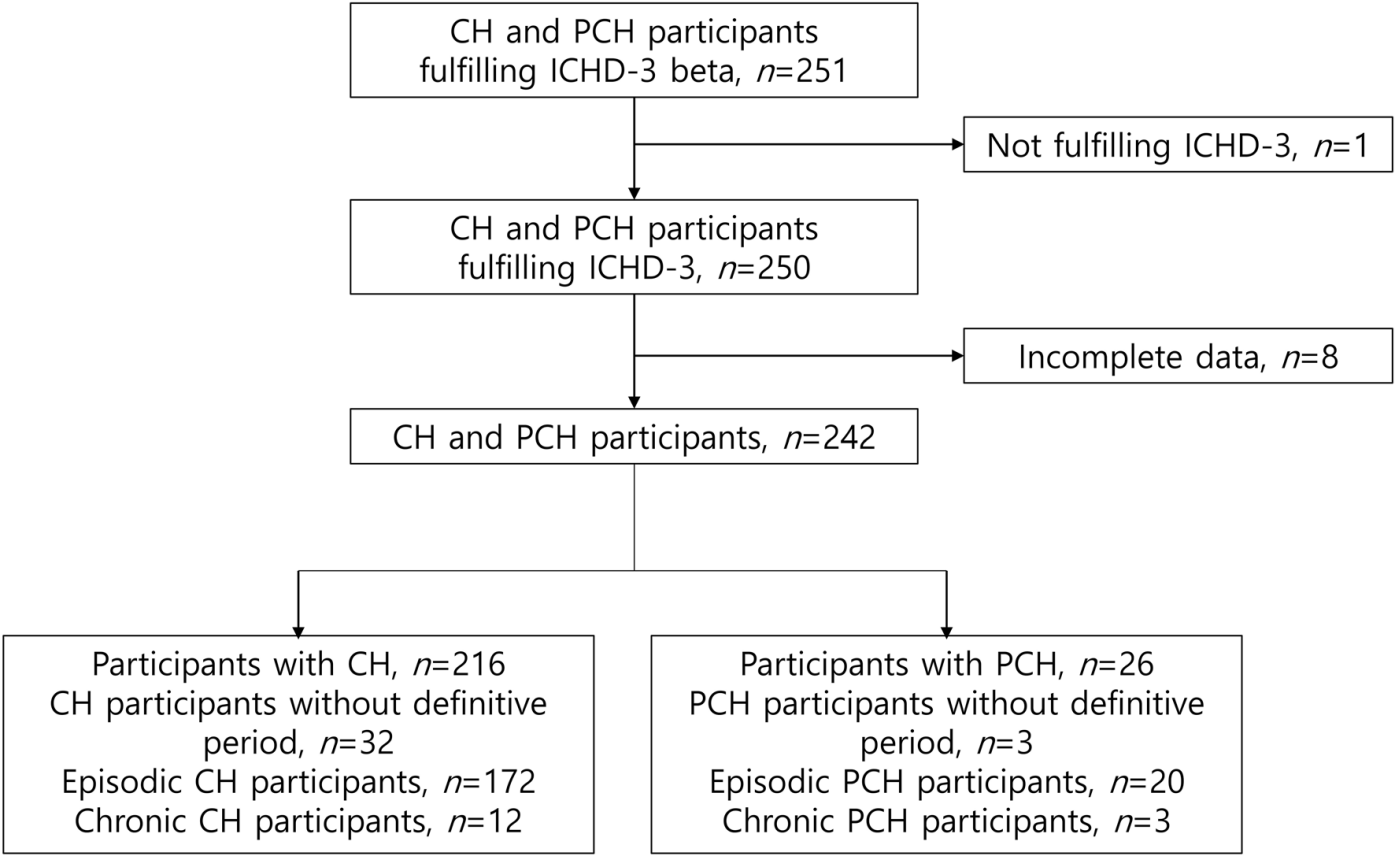
Flow diagram of participant selection. *CH* cluster headache, *ICHD-3* The third edition of the International Classification of Headache Disorders, *ICHD-3 beta* The third edition beta version of the International Classification of Headache Disorders, *PCH* probable cluster headache.

Detailed information about the KCHR has been described previously^5,10^. In the KCHR, participants fulfilling the available diagnostic criteria for CH and PCH, the third edition beta version of the International Classification of Headache Disorders (ICHD-3 beta), at the time it was created were enrolled^11^. For the present study, we included participants fulfilling the diagnostic criteria for CH and PCH based on ICHD-3^2^. A flow diagram of the participant selection process is presented in Fig. 1.

The KCHR collected data on the following parameters for all participants: sex, age at onset of CH, height, weight, headache intensity on numeric rating scale (from 0 to 10), CH attack frequency per day, mean CH duration since the first cluster period, cluster period duration during the ictal period, total number of cluster periods, smoking status, impact of headache (Headache Impact Test-6 score), circadian and circannual rhythmicity of headache attacks, quality of life (the 3-level version of EuroQol five-dimension scale [EQ-5D-3L]), anxiety (Generalized Anxiety Disorder [GAD-7] score), and depression (Patient’s Health Questionnaire-9 [PHQ-9] score)^12,13^. Participants with a GAD-7 score of ≥ 10 were classified as having anxiety, while participants with a PHQ-9 score of ≥ 10 were classified as having depression^12,14^. The previously validated Korean versions of PHQ-9, GAD-7 and EQ-5D-3L were used^14–16^. The cluster period was designated as CH attacks occurring in a series for weeks to months, separated by remission periods, usually lasting months or years. The cluster period duration was defined as the duration between the first and last days of each cluster period. The total number of cluster periods was defined as the total number of cluster periods diagnosed as CH. Migraine history was evaluated in all participants. Investigators assessed the circadian and circannual rhythmicity of headache attacks by asking participants whether the CH attacks in cluster periods tended to occur at the same time of the day and year, respectively.

### Diagnosis of CH and PCH

The diagnoses of CH and PCH were based on the ICHD-3 criteria^2^. We further classified participants into one of the following groups based on the cluster period duration: no definitive period, episodic, or chronic. Participants whose first episode of headache attacks fulfilled the CH or PCH criteria were classified, respectively, as having CH or PCH without a definitive period. Participants whose CH or PCH attacks lasted at least 1 year without a remission period or with remissions lasting < 3 months were classified as having chronic CH (CCH) or PCH, respectively. Participants whose CH or PCH attacks occurred in cluster periods, in whom two or more cluster periods lasted from 7 days to 1 year when untreated, and in whom cluster periods were separated by pain-free remission periods of ≥ 3 months were classified as having episodic CH (ECH) or episodic PCH.

### Case definitions of CH and PCH without CAS

According to the ICHD-3, we evaluated CAS in participants with CH and PCH based on the presence of the following conditions: (1) conjunctival injection and/or lacrimation, (2) nasal congestion and/or rhinorrhoea, (3) eyelid oedema, (4) forehead and facial sweating, and (5) miosis and/or ptosis. Since our study started in September 2016, when only the ICHD-3 beta was available, we also assessed the presence of (1) a sensation of fullness in the ear and (2) forehead and facial flushing, which were included as CAS in the ICHD-3 beta but not in the ICHD-3^2,11^.

### Ethical considerations

The institutional review boards of the 16 participating hospitals (Severance Hospital, Bundang Jesaeng General Hospital, Uijeongbu St.Mary’s Hospital, Chuncheon Sacred Heart Hospital, Samsung Medical Center, Eulji Hospital, Seoul Medical Center, Presbyterian Medical Center, Ewha Womans University Mokdong Hospital, Kangbuk Samsung Hospital, Korea University Kuro Hospital, Seoul St. Mary’s Hospital, Gyeongsang National University Hospital, Chung-Ang University Hospital, Seoul Paik Hospital, and Dongtan Sacred Heart Hospital) approved this study. All participants provided written informed consent before study enrolment. All clinical investigations were conducted following the principles expressed in the Declaration of Helsinki and its following amendments.

### Statistical analyses

The Shapiro–Wilks test was used to evaluate the normality of data distribution. When a normal distribution was confirmed, Student’s *t* test was used to compare continuous variables. If a normal distribution was not confirmed, the Mann–Whitney *U* test was used, and values were expressed as medians (interquartile ranges). The chi-squared test was used to evaluate categorical variables. If an expected value was less than five, Fisher’s exact test was used instead of the chi-squared test. Results were considered statistically significant when two-tailed *p* values were less than 0.05. The sample size was based on the available data. Statistical analyses were performed using IBM SPSS Statistics for Windows, Version 23.0 (IBM Corp., Armonk, NY, USA).

## Results

### Participants

A total of 251 participants were enrolled. One participant was excluded for not fulfilling the ICHD-3 criteria, and eight were excluded due to incomplete data. Finally, 216 participants with CH and 26 participants with PCH were included in this study (Fig. 1). The reasons for a diagnosis of PCH were as follows: an atypical attack duration (n = 8, 30.7%), where the duration was longer (n = 6) or shorter (n = 1) than typical; missing accompanying symptoms (n = 6, 23.1%); < 5 total number of attacks (n = 5, 19.2%); < 1 attack every other day (n = 3, 11.5%); not a severe headache intensity (n = 2, 7.7%); and a bilateral pain location (n = 2, 7.7%). None of the participants with CH or PCH without CAS had a sensation of fullness in the ear or forehead and facial flushing, which were included as CAS in ICHD-3 beta^11^.

The frequencies of CAS in participants with CH and PCH are summarised in Table 1. Conjunctival injection and/or lacrimation was the most common CAS in participants with CH, followed by nasal congestion and/or rhinorrhoea. Sensation of fullness in the ear was the least common CAS, and ptosis or miosis was the second less common CAS. Similar patterns were seen in participants with PCH, with conjunctival injection and/or lacrimation being the most common CAS and ptosis or miosis being the least common.

**Table 1:** Distribution of accompanying symptoms in the participants.

Accompanying symptoms	All participants with CH or PCH *n* = 242, *n* (%)	CH (code 3.1, 3.1.1, and 3.1.2) *n* = 216, *n* (%)	ECH (code 3.1.1) *n* = 172, *n* (%)	CCH (code 3.1.2) *n* = 12, *n* (%)	PCH (code 3.5.1) *n* = 26, *n* (%)
**Cranial autonomic symptoms**					
Conjunctival injection and/or lacrimation	202 (83.5)	187 (86.6)	151 (87.8)	11 (91.7)	15 (57.7)
Nasal congestion and/or rhinorrhoea	134 (55.4)	123 (56.9)	110 (64.0)	3 (25.0)	11 (42.3)
Eyelid oedema	64 (26.4)	60 (27.8)	50 (29.1)	3 (25.0)	4 (15.4)
Forehead and facial sweating	68 (28.1)	66 (30.6)	54 (31.4)	5 (41.7)	2 (7.7)
Miosis and/or ptosis	50 (20.7)	48 (22.2)	39 (22.7)	2 (13.3)	2 (7.7)
Forehead and facial flushing	38 (15.7)	37 (17.1)	34 (19.8)	1 (8.3)	1 (3.8)
Sensation of fullness in the ear	22 (9.1)	20 (9.3)	19 (11.0)	0 (0.0)	2 (7.7)
**Non-cranial autonomic symptom**					
A sense of restlessness or agitation	116 (47.9)	107 (49.5)	85 (49.4)	8 (66.7)	9 (34.6)
Participants without cranial autonomic symptoms	26 (10.7)	19 (8.8)	15 (8.7)	1 (8.3)	7 (26.9)

*CH* cluster headache, *PCH* probable cluster headache, *ECH* episodic cluster headache, *CCH* chronic cluster headache.

### Clinical features of all participants according to the presence of CAS

The clinical features of all 242 participants are summarised in Table 2. The headache intensity was less severe in participants without CAS than in those with CAS. Anxiety (GAD-7 score ≥ 10) and depression (PHQ-9 score ≥ 10) were less prevalent in participants without CAS than in those with CAS. All other features were similar in participants with and without CAS.

**Table 2:** Clinical features of participants with CH and PCH according to the presence of CAS.

	Participants with CH and PCH *n* = 242, *n* (%)	Participants with CH and PCH with CAS *n* = 216, *n* (%)	Participants with CH and PCH Without CAS *n* = 26, *n* (%)	*p* value
Female, n (%)	37 (15.3)	33 (15.3)	4 (15.4)	1.000
Age (year), median and IQR	37 (30.0–44.0)	37.0 (30.3–43.8)	37.0 (28.8–44.3)	0.822
Height (cm), median and IQR	174.0 (169.0–178.0)	174.0 (169.3–178.0)	172.0 (168.8–175.3)	0.095
Weight (Kg), median and IQR	72.0 (64.8–79.3)	72.0 (64.0–79.8)	70.5 (65.8–77.8)	0.421
Attack frequency per day, median and IQR	1.4 (1.0–3.0)	1.5 (1.0–3.0)	1.0 (1.0–2.1)	0.686
Headache intensity (numeric rating scale), median and IQR	9.0 (8.0–9.0)	9.0 (8.0–10.0)	8.3 (8.0–10.0)	0.303
Attack duration (minutes), median and IQR	80.0 (60.0–120.0)	77.5 (60.0–120.0)	90.0 (60.0–120.0)	0.796
Migraine history, n (%)	33 (13.6)	30 (14.30)	3 (11.5)	1.000
Circadian rhythmicity in headache attacks, n (%)	134 (55.4)	121 (56.0)	13 (50.0)	0.560
Circannual rhythmicity in headache attacks, n (%)	219 (90.5)	198 (91.7)	21 (80.8)	0.073
Current smoking, n (%)	106 (43.8)	99 (45.8)	7 (26.9)	0.066
**Location of pain**				
Orbital, n (%)	193 (79.8)	174 (80.6)	19 (73.1)	0.370
Supraorbital, n (%)	121 (50.0)	112 (51.9)	9 (34.6)	0.097
Temporal, n (%)	138 (57.0)	121 (56.0)	17 (65.4)	0.362
Headache Impact Test-6 score, median and IQR	69.0 (63.0–75.0)	69.0 (63.0–75.0)	67.5 (57.8–72.8)	0.259
Anxiety (Generalized Anxiety Disorder-7 score), median and IQR	7.0 (3.0–11.0)	7.0 (3.0–12.0)	2.0 (1.0–7.5)	0.001
Generalized Anxiety Disorder-7 score ≥ 10, n (%)	87 (36.0)	83 (38.8)	4 (15.4)	0.018
Depression (Patient Health Questionnaire-9 score), median and IQR	6.0 (3.0–11.0)	7.0 (3.0–11.0)	4.0 (2.0–7.3)	0.029
Patient Health Questionnaire-9 score ≥ 10, n (%)	80 (33.1)	76 (35.3)	4 (15.4)	0.047
Quality of life (EQ-5D-3L), median and IQR	0.913 (0.819–1.000)	0.907 (0.774–1.000)	0.913 (0.868–1.000)	0.248

*CAS* cranial autonomic symptoms, *CH* cluster headache, *EQ-5D-3L* the 3-level version of EuroQol five-dimension scale, *IQR* interquartile range, *PCH* probable cluster headache.

### Clinical features of participants with CH based on the presence of CAS

Of the 216 participants with CH, 19 (8.8%) did not have CAS. Anxiety and depression were less severe in participants with CH without CAS than in those with CAS. Other characteristics were not associated with the presence of CAS (Table 3).

**Table 3:** Clinical features of participants with CH according to the presence of CAS.

	Participants with CH *n* = 216	Participants with CH with CAS *n* = 197	Participants with CH without CAS *n* = 19	*p* value
Female, n (%)	30 (13.9)	29 (14.7)	1 (5.3)	0.484
Age (year), n (%)	37.0 (30.0–44.0)	37.0 (30.0–43.5)	38.0 (25.0–45.0)	0.933
Height (cm), median and IQR	173.5 (170.0–178.0)	174.0 (170.0–178.0)	172.0 (169.0–176.0)	0.376
Weight (Kg), median and IQR	72.0 (64.3–79.0)	72.0 (64.0–79.0)	71.0 (66.0–80.0)	0.879
Attack frequency per day, median and IQR	1.5 (1.0–3.0)	1.5 (1.0–3.0)	1.5 (1.0–2.5)	0.686
Headache intensity (numeric rating scale), median and IQR	9.0 (8.0–10.0)	9.0 (8.0–10.0)	9.0 (8.0–10.0)	0.303
Attack duration (minutes), median and IQR	90.0 (60.0–120.0)	90.0 (60.0–120.0)	120.0 (60.0–120.0)	0.581
Migraine history, n (%)	28 (13.0)	27 (13.7)	1 (5.3)	0.479
Circadian rhythmicity in headache attacks, n (%)	125 (57.9)	116 (59.5)	9 (47.4)	0.306
Circannual rhythmicity in headache attacks, n (%)	197 (91.2)	181 (92.3)	16 (84.2)	0.221
Current smoking, n (%)	98 (45.4)	91 (46.2)	7 (36.8)	0.434
**Location of pain**				
Orbital, n (%)	172 (79.6)	158 (80.2)	14 (73.7)	0.500
Supraorbital, n (%)	109 (50.5)	102 (51.8)	7 (36.8)	0.214
Temporal, n (%)	120 (55.6)	110 (55.8)	10 (52.6)	0.788
Headache Impact Test-6 score, median and IQR	69.0 (63.0–75.0)	69.0 (63.0–75.0)	70.0 (64.0–76.0)	0.938
Anxiety (Generalized Anxiety Disorder-7 score), median and IQR	7.0 (3.0–11.75)	8.0 (3.0–12.0)	2.0 (1.0–6.0)	0.002
Generalized Anxiety Disorder-7 score ≥ 10, n (%)	81 (37.5)	78 (39.6)	3 (15.8)	0.048
Depression (Patient Health Questionnaire-9 score), median and IQR	6.0 (3.0–11.0)	7.0 (3.0–11.0)	3.0 (1.0–7.0)	0.030
Patient Health Questionnaire-9 score ≥ 10, n (%)	71 (32.9)	68 (34.5)	3 (15.8)	0.126
Quality of life (EQ-5D-3L), median and IQR	0.907 (0.819–1.000)	0.903 (0.785–1.000)	0.913 (0.870–1.000)	0.272

*CAS* cranial autonomic symptoms, *CH* cluster headache, *EQ-5D-3L* the 3-level version of EuroQol five-dimension scale, *IQR* interquartile range.

Among the 172 participants with ECH, 15 (8.7%) did not have CAS. Anxiety and depression were less prevalent in participants with ECH without CAS than in those with CAS. The average cluster period duration in months was longer in those with ECH without CAS than in those with CAS. Other clinical features were similar between participants with ECH with and without CAS (Table 4). Of the 12 participants with CCH, two (16.7%) did not have CAS. The frequencies of the absence of CAS in ECH and CCH were similar (8.7% [15/172] vs 16.7% [2/12], *p* = 0.612).

**Table 4:** Clinical features of participants with ECH according to the presence of CAS.

	Participants with ECH *n* = 172	Participants with ECH with CAS *n* = 157	Participants with ECH without CAS *n* = 15	*p* value
Female, n (%)	22 (12.8)	21 (13.4)	1 (6.7)	0.696
Age (year), median and IQR	37 (30.25–44.0)	37.0 (31.5–44.0)	38.0 (24.0–44.0)	0.565
Onset age (year), median and IQR	10.0 (5.0–16.0)	10.0 (5.0–16.0)	60. (2.0–10.0)	0.067
Height (cm), median and IQR	174.0 (170.0–178.0)	175.0 (17.0–178.0)	172.0 (169.0–177.0)	0.305
Weight (Kg), median and IQR	72.0 (65.0–79.0)	72.0 (64.0–79.0)	70.0 (66.0–80.0)	0.698
Cluster duration (year), median and IQR	7.0 (3.0–12.0)	7.0 (3.0–12.0)	7.0 (2.0–10.0)	0.263
Total number of cluster periods, median and IQR	7.0 (3.0–12.0)	7.0 (3.0–12.0)	7.0 (2.0–10.0)	0.263
Attack frequency per day, median and IQR	1.4 (1.0–3.0)	1.5 (1.0–3.0)	1.0 (1.0–2.0)	0.383
Headache intensity (numeric rating scale), median and IQR	9.5 (8.0–10.0)	10.0 (8.5–10.0)	9.0 (8.0–10.0)	0.153
Attack duration (minutes), median and IQR	60.0 (60.0–120.0)	60.0 (60.0–120.0)	90.0 (35.0–120.0)	0.688
Average cluster period duration (month), median and IQR	4.0 (3.0–8.0)	4.0 (3.0–7.0)	6.0 (4.0–10.0)	0.020
Average remission period (month), median and IQR	12.0 (10.0–24.0)	12.0 (10.0–24.0)	12.0 (6.0–12.0)	0.502
Regular attack pattern, n (%)	121 (70.3)	113 (72.0)	8 (53.3)	0.131
Migraine history, n (%)	17 (9.9)	17 (11.0)	0 (0.0)	0.368
Circadian rhythmicity in headache attacks, n (%)	103 (59.9)	96 (61.1)	7 (46.7)	0.426
Circannual rhythmicity in headache attacks, n (%)	170 (98.8)	155 (98.7)	15 (100.0)	1.000
Current smoking, n (%)	80 (46.5)	75 (47.8)	5 (33.3)	0.417
**Location of pain**				
Orbital, n (%)	137 (79.7)	127 (80.9)	10 (66.7)	0.191
Supraorbital, n (%)	84 (48.8)	78 (49.7)	6 (40.0)	0.474
Temporal, n (%)	92 (53.5)	84 (53.5)	8 (53.3)	0.990
Headache Impact Test-6 score, median and IQR	69.0 (63.0–75.0)	69.0 (63.0–75.0)	70.0 (64.0–76.0)	0.938
Anxiety (Generalized Anxiety Disorder-7 score), median and IQR	7.0 (3.0–11.8)	8.0 (3.0–12.0)	2.0 (1.0–6.0)	0.001
Generalized Anxiety Disorder-7 score ≥ 10, n (%)	81 (37.5)	78 (39.6)	3 (15.8)	0.050
Depression (Patient Health Questionnaire-9 score), median and IQR	6.0 (3.0–11.0)	7.0 (3.0–11.0)	3.0 (1.0–7.0)	0.036
Patient Health Questionnaire-9 score ≥ 10, n (%)	71 (32.9)	68 (34.5)	3 (15.8)	0.040
Quality of life (EQ-5D-3L), median and IQR	0.913 (0.819–1.000)	0.913 (0.819–1.000)	0.913 (0.907–1.000)	0.087

*CAS* cranial autonomic symptoms, *ECH* episodic cluster headache, *EQ-5D-3L* the 3-level version of EuroQol five-dimension scale, *IQR* interquartile range.

### Clinical features of participants with PCH based on the presence of CAS

Of the 26 participants with PCH, 7 (26.9%) did not have CAS. Headache intensity was less severe in participants with PCH without CAS than in those with CAS. The prevalence of anxiety and depression and other clinical features were similar in participants with PCH with and without CAS (Table 5). The frequency of not having CAS was higher in participants with PCH than in those with CH (26.9% [7/26] vs 8.7% [19/216], *p* = 0.005).

**Table 5:** Clinical features of participants with PCH according to the presence of CAS.

	Participants with PCH *n* = 26	Participants with PCH with CAS *n* = 19	Participants with PCH without CAS *n* = 7	*p* value
Female, n (%)	7 (26.9)	4 (21.1)	3 (42.9)	0.340
Age (year), median and IQR	38.5 (33.8–43.3)	40.0 (35.0–44.0)	36.0 (30–43.0)	0.534
Onset age (year), median and IQR	32.0 (28.8–42.3)	32.0 (28.0–42.0)	36.0 (29.0–43.0)	0.572
Height (cm), median and IQR	174.5 (164.8–177.5)	175.0 (165.0–180.0)	169.0 (158.0–175.0)	0.188
Weight (Kg), median and IQR	72.0 (66.0–81.0)	73.0 (68.0–84.0)	69.0 (55.0–72.0)	0.231
Cluster duration (year), median and IQR	1.0 (0.0–6.3)	2.0 (0.0–9.0)	1.0 (0.0–3.0)	0.427
Total number of cluster periods, median and IQR	1.5 (1.0–4.0)	2.0 (1.0–4.0)	1.0 (1.0–2.0)	0.534
Attack frequency per day, median and IQR	1.0 (1.0–2.0)	1.0 (1.0–3.0)	1.0 (1.0–2.0)	0.778
Headache intensity (numeric rating scale), median and IQR	8.5 (7.8–10.0)	9.5 (8.0–10.0)	8.0 (7.0–8.0)	0.015
Attack duration (minutes), median and IQR	60.0 (35.0–180.0)	60.0 (30.0–195.0)	60.0 (60.0–120.0)	0.790
Average cluster period duration (month), median and IQR	3.5 (2.3–4.0)	4.0 (2.0–4.0)	3.0 (2.0–3.0)	0.727
Average remission period (month), median and IQR	23.0 (5.0–29.0)	23.0 (4.0–28.5)	12.0 (6.0–12.0)	> 0.999
Regular attack pattern, n (%)	6 (23.1)	5 (26.3)	1 (14.3)	> 0.999
Migraine history, n (%)	5 (19.2)	3 (15.8)	2 (28.6)	0.588
Circadian rhythm, n (%)	9 (34.6)	5 (27.8)	4 (571)	0.205
Circannual rhythm, n (%)	22 (84.6)	1 (5.6)	2 (28.6)	0.180
Current smoking, n (%)	8 (30.8)	8 (42.1)	0 (0.0)	0.062
**Location of pain**				
Orbital, n (%)	21 (80.8)	16 (84.2)	5 (71.4)	0.588
Supraorbital, n (%)	12 (46.2)	10 (52.6)	2 (28.6)	0.391
Temporal, n (%)	18 (69.2)	11 (57.9)	7 (100.0)	0.062
Headache Impact Test-6 score, median and IQR	65.5 (57.0–72.0)	67.0 (58.0–76.0)	58.0 (50.0–68.0)	0.107
Anxiety (Generalized Anxiety Disorder-7 score), median and IQR	5.0 (2.0–9.8)	5.0 (3.0–11.0)	4.0 (3.0–8.0)	0.455
Generalized Anxiety Disorder-7 score ≥ 10, n (%)	6 (23.1)	5 (26.3)	1 (14.3)	> 0.999
Depression (Patient Health Questionnaire-9 score), median and IQR	4.0 (3.5–13.0)	5.5 (3.8–13.8)	4.0 (3.0–8.0)	0.495
Patient Health Questionnaire-9 score ≥ 10, n (%)	9 (34.6)	8 (42.1)	1 (14.3)	0.357
Quality of life (EQ-5D-3L), median and IQR	0.913 (0.833–1.000)	0.913 (0.749–1.000)	0.899 (0.862–0.913)	0.427

*CAS* cranial autonomic symptoms, *EQ-5D-3L* the 3-level version of EuroQol five-dimension scale, *PCH* probable cluster headache, *IQR* interquartile range.

## Discussion

The main findings of the present study were as follows: (1) Approximately one-eleventh of participants with CH and a quarter of those with PCH did not have CAS; (2) Anxiety and depression were less severe in participants with CH without CAS than in those with CAS; and (3) Headache intensity was milder in participants with PCH without CAS than in those with CAS. Other clinical features of CH and PCH did not differ between participants with and without CAS.

CH has been characterised by recurrent attacks of severe unilateral headache and ipsilateral CAS^1,17^. CAS were considered a key characteristic of CH and included as a diagnostic criterion since the publishing of the first edition of the ICHD^8,18^. Nevertheless, it has been reported that some individuals with CH-like headaches did not experience CAS during headache attacks^9,19^. The second edition of the ICHD, published in 2004, included a sense of restlessness or agitation as an accompanying symptom of CH, in addition to CAS, and a diagnosis of CH without CAS became possible^4^. The subsequent editions of diagnostic criteria for CH retained restlessness or agitation as an accompanying symptom along with CAS^2,11^. Therefore, CH and PCH without CAS are currently included in the ICHD-3, and our study enrolled participants based on these definitions.

We found that 8.8% of participants with CH did not have CAS. This frequency was similar or somewhat higher than that found in previous studies. A case series of 163 patients with CH in Sweden found that 3.1% of the patients did not have CAS^19^, while an Italian clinic-based study of 251 patients with CH found that 2.8% of the patients did not have CAS^8^. Another Italian study found that 7.5% of patients with CH did not have CAS^9^. In a clinic-based study in Portugal, not having CAS was reported in 6.1% of patients with CH or PCH^7^. Possible causes for the discrepancy between the findings from our study and those from previous studies include differences in the diagnostic criteria, ethnicity, and study setting. The abovementioned Swedish study used three criteria for CH diagnosis (World Federation of Neurology, Ekbom, and ICHD-1)^18,20,21^. The two Italian studies used the ICHD-1, which did not include restlessness and agitation as accompanying symptoms. All four studies were conducted in European countries, while our study was conducted in Korea. Individuals with CH in Asian countries showed lower attack frequencies and shorter attack durations than those in Western countries^22^. All four previous studies used data from a single hospital, while the present study used data from the KCHR, which contained data from 16 hospitals.

It has been consistently reported that individuals with CH have a higher frequency of anxiety and depression than those with migraine or without headache^10,23–27^. Additionally, it has been demonstrated that individuals with CCH are more likely to be affected by anxiety and depression than those with ECH^10^. High frequencies of anxiety and depression were observed in the CH and PCH groups in the present study. Furthermore, to the best of our knowledge, this study is the first to report that anxiety and depression were less severe in participants with CH without CAS than in those with CAS. What is the possible mechanism underlying the association of anxiety and depression with CAS? One possible explanation is the role of shared anatomical substrates between the affective symptoms and CH in the pathogenesis of CAS. Neuroimaging findings have revealed an altered metabolism of the pain matrix and hypothalamus in patients with CH and affective disorders^28–30^. Therefore, decreased frequencies of anxiety and depression in participants with CH without CAS may be related to the roles of the pain matrix and hypothalamus in the pathogenesis of CAS. This hypothesis could be evaluated by comparing the neuroimaging findings between individuals with CH with and without CAS.

Parasympathetic activation mediated by the trigeminal-autonomic reflex has been ascertained as the mechanism for the presentation of CAS in CH^31^. The trigeminal-autonomic reflex is a brainstem connection between the trigeminal nerve and facial cranial parasympathetic nerve outflow, which is activated by the stimulation of the trigeminovascular system^32^. The hypothalamus has been hypothesised to play a role in initiating CH attacks and causing the activation of the trigeminovascular system^33,34^. Once triggered, this system stimulates the trigeminal-autonomic reflex and results in the occurrence of CAS^35^. The present study found that approximately 9% of individuals with CH did not have CAS; these individuals had less anxiety and depression, which are closely related with the hypothalamus, compared to those with CAS. These findings suggest that some individuals with CH may have a weaker signal from the hypothalamus, which causes headache attacks but does not produce CAS. The weaker signal induces less anxiety and depression in individuals with CH. Our findings also suggest that the activation of the trigeminal-autonomic reflex is not an essential part of CH attacks.

Conjunctival injection and/or lacrimation has been consistently reported to be the most common CAS in individuals with CH^8,36–38^. In the present study, conjunctival injection and/or lacrimation, as the most frequent CAS, was observed in 86.6% of participants with CH. Conjunctival injection and/or lacrimation was the most frequent CAS in Asian as well as Western countries^6,22,39,40^. The frequency of CAS in the present study was similar to that found in previous studies from Asian countries. The frequency of ptosis or miosis in the present study (22.2%) was lower than that found in studies from Western countries. A prospective clinical study in the UK found that 76% of individuals with CH had ptosis^38^. A Danish study reported that ptosis was present in 44.8% of patients with CH^37^. In Asian studies, a lower frequency of ptosis or miosis has been observed—a Japanese study showed a ptosis frequency of 8.1%^6^, while a study at a Chinese tertiary headache centre revealed that ptosis or miosis was present in 16.7% of patients with CH^40^ and a Korean multicentre study reported a rate of 8.5%^39^. The similarity in CAS frequencies mentioned above suggests that this parameter was correctly evaluated in the present study.

Our study had some limitations. First, it was conducted as a multicentre prospective study, but it did not represent the whole CH and PCH population. Therefore, our findings should be validated using another dataset to enhance the generalisability. Second, we included relatively small samples of patients with PCH and CCH. Although we tried to enrol all eligible patients with PCH and CCH over the 3-year study period, the number of relevant cases may have been too small for some subgroup analyses. In other words, the statistical power was weakened by the limited sample size. Further studies that include a sufficient number of participants with PCH and CCH are needed to verify our findings. Third, we used the GAD-7 and PHQ-9 to assess anxiety and depression, respectively. However, these instruments only indicate a state of anxiety and depression and cannot be used to confirm the diagnosis. Additional analyses diagnosing anxiety and depression according to the fifth edition of the Diagnostic and Statistical Manual of Mental Disorders will confirm the relationship of anxiety and depression with CAS in CH.

In conclusion, nearly 9% of participants with CH did not have CAS. The prevalence of anxiety and depression was lower in these patients than in those with CAS. Other clinical features were similar between participants with and without CAS. One-quarter of the participants with PCH did not have CAS and headache intensity was less severe in these participants than in those with CAS. The frequency of anxiety and depression did not differ between participants with PCH with and without CAS. Our study proposed that some clinical features of CH and PCH differed based on the presence of CAS. The findings of the present study may help enhance the understanding of the pathophysiology of CH.

## Data Availability

The data used in the present study are available from the corresponding author on reasonable request.
